# VitiCanopy: A Free Computer App to Estimate Canopy Vigor and Porosity for Grapevine

**DOI:** 10.3390/s16040585

**Published:** 2016-04-23

**Authors:** Roberta De Bei, Sigfredo Fuentes, Matthew Gilliham, Steve Tyerman, Everard Edwards, Nicolò Bianchini, Jason Smith, Cassandra Collins

**Affiliations:** 1 School of Agriculture, Food and Wine, Waite Research Institute, the University of Adelaide, PMB 1 Glen Osmond 5064, South Australia, Australia; roberta.debei@adelaide.edu.au (R.D.B.); matthew.gilliham@adelaide.edu.au (M.G.); stephen.tyerman@adelaide.edu.au (S.T.); 2 Faculty of Veterinary and Agricultural Sciences, the University of Melbourne, Parkville 3010, Victoria, Australia; sigfredo.fuentes@unimelb.edu.au; 3 ARC Centre of Excellence in Plant Energy Biology, Waite Research Institute, PMB 1 Glen Osmond 5064, South Australia, Australia; 4 CSIRO Agriculture, Waite Campus Laboratory, Private Bag 2, Glen Osmond 5064, South Australia, Australia; Everard.Edwards@csiro.au (E.E.); nicolo.bianchini@gmail.com (N.B.); 5 Dipartimento di Scienze Agrarie (DipSA), the University of Bologna, Area Colture Arboree, Viale Fanin 46, 40127 Bologna, Italy; 6 National Wine and Grape Industry Centre, Charles Sturt University, Locked Bag 588, Wagga Wagga 2678, New South Wales, Australia; Jason.Smith@hs-gm.de

**Keywords:** canopy vigor, LAI, PAI, computer application, light extinction coefficient, image analysis, cover photography

## Abstract

Leaf area index (LAI) and plant area index (PAI) are common and important biophysical parameters used to estimate agronomical variables such as canopy growth, light interception and water requirements of plants and trees. LAI can be either measured directly using destructive methods or indirectly using dedicated and expensive instrumentation, both of which require a high level of know-how to operate equipment, handle data and interpret results. Recently, a novel smartphone and tablet PC application, VitiCanopy, has been developed by a group of researchers from the University of Adelaide and the University of Melbourne, to estimate grapevine canopy size (LAI and PAI), canopy porosity, canopy cover and clumping index. VitiCanopy uses the front in-built camera and GPS capabilities of smartphones and tablet PCs to automatically implement image analysis algorithms on upward-looking digital images of canopies and calculates relevant canopy architecture parameters. Results from the use of VitiCanopy on grapevines correlated well with traditional methods to measure/estimate LAI and PAI. Like other indirect methods, VitiCanopy does not distinguish between leaf and non-leaf material but it was demonstrated that the non-leaf material could be extracted from the results, if needed, to increase accuracy. VitiCanopy is an accurate, user-friendly and free alternative to current techniques used by scientists and viticultural practitioners to assess the dynamics of LAI, PAI and canopy architecture in vineyards, and has the potential to be adapted for use on other plants.

## 1. Introduction

Monitoring grapevine canopy architecture is critical for the assessment of growth, vigor and light transmission through the canopy and water requirements of plants. Furthermore, canopy architecture parameters can be correlated to yield, grape quality and the potential productivity of future seasons [1,2,3].

One of the most important factors in the assessment of grapevine canopies is the leaf area index (LAI), which is commonly defined as the total one-sided area of leaf tissue per unit ground surface area [4]. Similarly, plant area index (PAI) is another indicator of canopy size and is defined as the sum of LAI and woody structures or non-leaf material [5]. Both LAI and PAI are difficult to measure or estimate since they require either destructive techniques (often used to measure LAI) that are labor intensive, or expensive instrumentation for indirect estimations (which measure PAI) [6].

Direct planimetric measurements can give very accurate LAI results, since they require stripping the whole canopy and scanning every leaf with a scanning planimeter (e.g., Li-3000/3100, Li-Cor, Lincoln, NE, USA) to obtain the total area of leaves per unit area of soil [7]. However, this method is extremely time consuming and destructive so it is of little value in a commercial setting other than for the calibration of non-destructive and indirect techniques [5,8]. Similarly, the gravimetric method is based on the correlation between leaf area and dry weight [5,7,9], which can be applied with a high percentage of accuracy. However, this technique is site and species/cultivar specific and yet another time-consuming and extremely labor intensive method to obtain accurate results [10]. Other methods that are widely applied in vineyards, use the relationship between leaf area and leaf length or shoot length [11,12]; these are common because they are simple, but nonetheless time consuming and season, site, climate and cultivar specific.

Indirect methods are based on instrumentation that measure light transmission through the canopy in the photosynthetic active radiation (PAR) wavelengths to estimate canopy size and vigor (e.g., SunSCAN, Delta-T Devices Ltd, Cambridge, UK; AccuPAR, Decagon Devices, Pullman, WA, USA). Others measure the gap fraction for different zenithal angles, such as the LAI-2000 and 2200 (Li-Cor, Lincoln, NE, USA). Indirect measures include all canopy elements (leaves and wood) in the estimation so the output is regarded as PAI rather than LAI [5]. There are advantages and disadvantages for all of these methods and they have been extensively outlined in the literature [5,6,7,9,13]. Indirect methods are generally faster/easier to automate, allowing for larger sampling volume. However, the dedicated instrumentation can be cost prohibitive and requires specific know-how for usage, data collection, downloading and interpretation of results. One of the most versatile and easy to use indirect methods to estimate canopy size, as identified in the literature, uses digital or cover photography, which requires the use of digital cameras; this method is based on image analysis algorithms that consider the pixel count corresponding to canopy material and the gap fraction [7,14,15]. Image analyses can be performed on a *post-hoc* basis using Adobe Photoshop [13,15] or using semi-automated and automated computer analysis software [6]. An automated method to estimate grapevine canopy size using digital photography and Matlab programming based on previously published works, has been proposed [15,16,17,18]. Fuentes *et al.* [16] and Poblete-Echeverría *et al.* [18] have demonstrated that cover photography and Matlab programming could be used to estimate canopy architecture parameters for grapevines and apple trees with results highly correlated to both destructive methods and those obtained using specialized instrumentations, such as the LAI-2000. This method, applied to the wine industry and agriculture in general, could fulfill the need to have long term monitoring of spatial and temporal dynamics of canopy growth and leaf area development throughout the season. However, it still requires a customized code in Matlab and computer skills to analyze the images, restricting usage to researchers or for management purposes within major agricultural industries. To make this technology more accessible to growers and the non-scientific community, the University of Adelaide, jointly with the University of Melbourne, has developed a smartphone and tablet PC App, called VitiCanopy, to estimate canopy architectural parameters. VitiCanopy is based on cover photography obtained at 0° Zenith angle and the Matlab method for image analysis proposed for grapevines [16,19]. The VitiCanopy App has been launched in its iPhone/iPad version (iOS) and the Android version has been developed and will soon be released. Another App to measure LAI, called PocketLAI, has been proposed for use on Android smartphones [20]. The latter methodology uses the device accelerometer and gap fraction calculations to derive LAI from images taken while rotating the device [21]. PocketLAI has been tested on various crops including rice, corn, natural grassland and tree canopies and compared to other commercial tools (e.g., LAI-2000 and ceptometer) with similar results [20,22,23]. However, it has not been implemented or tested for grapevines and, until the date of this paper, it is not available for download in the Google Play Store.

This paper describes the full development, beta testing and validation of VitiCanopy, a novel App for smartphones and tablet PCs to estimate canopy size, vigor, canopy porosity and other architectural parameters for grapevine. VitiCanopy, as all other indirect methods, does not distinguish between leaf and non-leaf material in the analysis; however, for consistency with previous literature on the cover photography method [15,16,17,18,24,25,26] and outputs from other commercial instrumentation [27] used in this work, the term LAI was adopted in this manuscript and LAI-vc refers to the output obtained with VitiCanopy.

## 2. Materials and Methods

### 2.1. VitiCanopy Development

**Figure 1:**
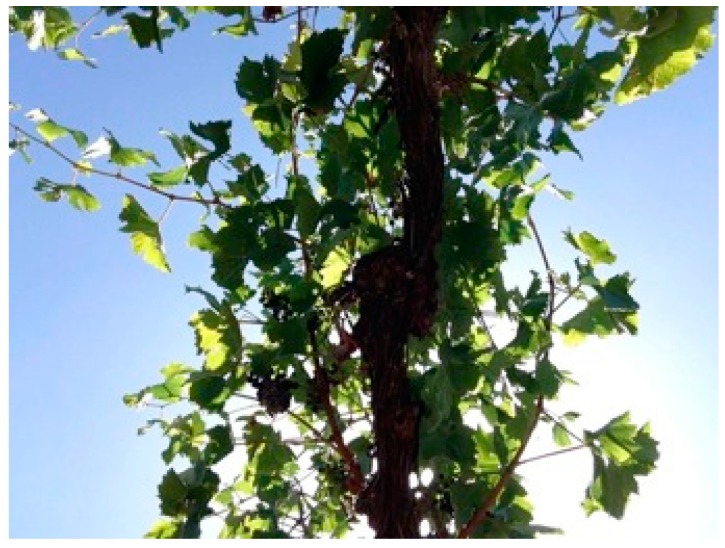
Example of an upward looking grapevine canopy image suitable for analysis using VitiCanopy. The image was obtained using the front camera of an iPad4, at a distance of 80 cm between the vine’s cordon and the device.

VitiCanopy, a program that can be downloaded to a smartphone or tablet device using iOS (Apple Inc., Cupertino, CA, USA), was developed using Xcode 7 and Objective-C. Some image processing (e.g., the real-time monochrome camera preview) uses a third party open source library called GPUImage, which takes advantage of the parallel processing capability of the phone’s GPU (Graphical Processing Unit) by running image processing algorithms as GPU shader programs. These images are automatically analyzed using the methodology and algorithms described in previous publications [6,16]. It is recommended that the images are taken using the front camera of the device to allow the operator to see the image to be taken on the screen and instantly judge its suitability for analysis. The use of a “selfie stick” for operator comfort is also recommended. Images can be obtained using the back camera and the App also allows the analysis of images previously obtained and stored in the camera files (camera roll for iPhones/iPads). Figure 1 is an example of a suitable image for analysis with VitiCanopy. This code has been since translated into Android Studio and SDK tools and libraries (Google Inc., Cupertino, CA, USA).

### 2.2. Algorithms Used in the VitiCanopy

VitiCanopy uses the following image analysis algorithms to calculate canopy architectural parameters, which are based on gap analysis from upward-looking images of canopies and the transmission of light though the canopy to estimate LAI-vc (which includes non-leaf material in the results) based on Beer’s Law [14,15]:

(1) $$f_{f}=1-\frac{\text{tg}}{\text{tp}}$$

(2) $$f_{c}=1-\frac{\text{lg}}{\text{tp}}$$

(3) $$\Phi =1-\frac{f_{f}}{f_{c}}$$

where lg = large gap pixels count; tg = total pixels in all gaps and tp = total pixels from the image; fractions of foliage projective cover (*f_f_*); crown cover (*f_c_*); crown porosity (Φ). The *f_f_* parameter has been defined as the “proportion of ground area covered by the vertical projection of foliage and branches” and Φ as the “proportion of the ground area covered by the vertical projection of foliage and branches within the perimeter of the crowns of individual plants” [15,28].

LAI-vc is then calculated from the Beer’s Law equation as:

(4) $$\text{LAIvc}=-f_{c}\frac{\text{ln}\phi }{k}$$

where k is the light extinction coefficient (0 > k >1).

The clumping index at the zenith angle, Ω(0), is calculated as follows:

(5) $$\Omega \left(0\right)=\frac{\left(1-\phi \right)\text{ln}\left(1-f_{f}\right)}{\text{ln}(\phi )/f_{f}}$$

The clumping index is a correction factor to obtain effective LAI-vc (LAI-vc_e_), which is the product of:

LAI-vc_e_ = LAI-vc × Ω(0) (6)

Specifically, the clumping index describes the non-random distribution of canopy elements. If Ω(0) = 1, it means that the canopy displays random dispersion; for 0 < Ω(0) < 1, the canopy is defined as clumped. Since grapevines are trained to obtain uniform and continuous rows, the value of this index is expected to be close to 1 or less clumped. Therefore, for grapevine, LAI-vc should be close to LAI-vc_e_. For the lg calculation, the App automatically generates a binary image and sub-divides this image into 25 sub-images. From each of these sub-images the App automatically counts the pixels corresponding to canopy (ones) and sky (zeros). The 5 × 5 subdivision has been shown to be the most effective to determine large gaps using the cover photography method requiring minimal computational power [6].

### 2.3. Description of VitiCanopy

**Figure 2:**
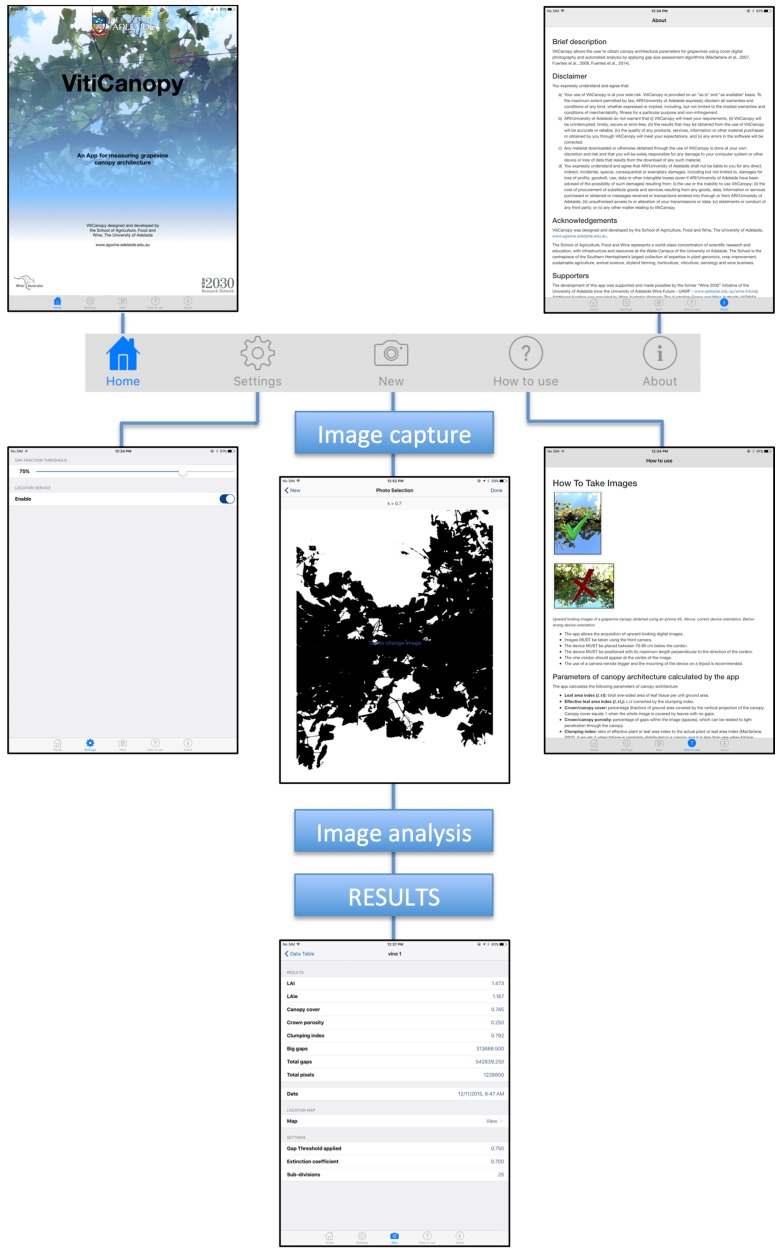
VitiCanopy Home page displaying the five main menu tabs as a simplified operations flow chart.

VitiCanopy, once installed and launched on the device, presents a “Home” page with a menu consisting of five icons (Figure 2). An instructional YouTube video guiding users through the process is available in [29]. The “Home” page contains the University of Adelaide and funding bodies’ logos, with a brief description of the App. The “Settings” menu allows the user to set up the gap fraction threshold (see below) and to enable/disable the location services linked to the GPS. The “New” menu allows the user to perform new measurements. The “How to Use” menu contains information on how to acquire and analyse images and a description of the algorithms used to calculate canopy parameters. This menu also has examples of suitable and unsuitable images; describing most common errors, such as capturing the sun in the image and the operator head in the frame, among others. The “About” menu contains: (i) a brief description of the capabilities of the App; (ii) disclaimer notice; (iii) acknowledgments; (iv) supporters; (v) development team; (vi) programmers; (vii) source code; (viii) contact information; and (ix) a list of references.

### 2.4. Settings Menu

The settings menu consists of two options that need to be defined by the user. The first setting option is the “GAP FRACTION THRESHOLD”. The gap fraction ranges from 0% to 100%. Generally, the gap fraction is set to 75%, meaning that if 75% of pixels from each of the sub-images (25 in total) correspond to sky, this sub-image is considered in the “large gap” (lg) count for the calculations in Equation (2).

The “LOCATION SERVICE” menu is used to enable/disable the GPS capabilities of the device within the App. By enabling this service, geo-location data will be recorded in the analysis and allows mapping data and spatial assessment within and between vineyards. Disabling the service does not disable the location services from the device’s settings.

### 2.5. New Measurement Menu

When performing a new measurement, the user needs to tap the “New” icon and then the “+” icon on the top right corner of the screen, a “FILE” name can be entered to identify every image taken. As default, when no name is entered, the date and time will be assigned to every image and analysis. The current “LOCATION” (latitude and longitude data from the GPS) is reported in this screen. If the “Not Available” message appears in this section, it means that the location service was either disabled or that there is no service at that particular location from the phone carrier provider.

By tapping the “PHOTO SELECTION” tab, the App will present options to either take a new image, or to choose an image from stored files (*i.e.*, the device’s camera roll). The file option allows the user to analyze digital images previously obtained. When the camera option is selected, a black and white image (binary) is automatically displayed, this allows the user to visually evaluate the suitability of the image for analysis and determine whether there are obvious errors from the use of the automated threshold, which can be subsequently altered. If an image is selected from files, the App will also show it as a binary image in order to judge its suitability.

Once the “PHOTO SELECTION” tab is chosen, the photo is displayed as a monochrome or binary image showing the light extinction coefficient (*k*), which is set at 0.7 by default, since this value has been described to be the most accurate for grapevine canopies [16] (more information provided in the discussion of this paper). However, if the user requires the use of a different value, the k can be adjusted.

### 2.6. Outputs

Once individual images have been acquired using the camera or loaded from files, the user presses “Done” and a pop up message is generated which shows LAI (in this paper equals LAI-vc_e_, which includes non-leaf material in the analysis) and canopy porosity. All the images collected are stored as RGB Joint Photographic Expert Group files (.JPG) in the camera roll for further analyses (or to re-visit the analysis if required) and to keep a record of measurements. All the processed information is stored in the App as a text file (.txt). By tapping on a single analysis output the “RESULTS” page opens up showing all the calculated parameters from the specific image: (i) calculated LAI (= LAI-vc in this paper) using Equation (4); (ii) effective LAI_e_ (= LAI-vc_e_ in this paper) calculated using Equation (6); (iii) Canopy cover (Equation (1)); (iv) Crown porosity (Equation (2)); (v) Clumping index (Equation (5)); (vi) Big gaps; (vii) Total gaps; (viii) Total pixels; (ix) Date and time for image acquisition. A “LOCATION MAP” is also available which shows the exact location of where the image was taken. When scrolling down in the same window a “SETTINGS” tab also shows the settings used for the analysis of the specific image.

All the analyses can then be sent as a .csv file attached to an email by tapping “Export” on the top left corner of the “Data table” page.

### 2.7. Beta Testing of VitiCanopy

The beta testing of VitiCanopy was conducted during the seasons 2013–14 and 2014–15 in different vineyards across South Australia, specifically described below. The testing included the comparison between: (i) VitiCanopy and Matlab for analysing images [16]; VitiCanopy and the Plant Canopy Analyser, LAI-2000, for estimating canopy size; (ii) VitiCanopy and a planimetric or allometric method for measuring LAI. Since it has been previously reported that, for apple trees, canopy size calculations using the cover photography method are very sensitive to the *k* value used in the analysis [18], we compared different *k* and proxy-*k* values to evaluate the PAI estimation performance and sensitivity for grapevine canopies.

#### 2.7.1. VitiCanopy *vs.* Matlab

Images (*n* = 113) obtained from different vintages, in different vineyards across South Australia and in days with different sky conditions were randomly selected and analyzed using both VitiCanopy and the automatic customized code developed in Matlab (The Mathworks Inc., Natick, MA, USA), which has been extensively described previously [16]. Images were taken at 70 to 80 cm from the cordon. The same analysis inputs were used for all images and applied to both methods: (i) number of subdivision = 5 (25 sub-images); (ii) a gap fraction threshold equal to 0.75; and, (iii) a common light extinction coefficient (*k*) = 0.7 [16].

#### 2.7.2. VitiCanopy *vs.* LAI-2000

Canopy size was measured in three Shiraz (*Vitis vinifera* L.) vineyards located at Langhorne Creek (South Australia, Australia), Hilltops (New South Wales, Australia) and Sunraysia (Victoria, Australia). Two of the vineyards, Langhorne Creek and Hilltops, were trained to a single wire, bi-lateral cordon, with the third, Sunraysia, trained to a two-wire, double bilateral cordon. All three sites were part of a management trial, which included various canopy management techniques and generated a range of canopy sizes at each site. Measurements using the LAI-2000 (Plant canopy analyser, Li-Cor Inc., Lincoln, NE, USA) and the VitiCanopy app were made in parallel.

The LAI-2000 was used with the view restricted to a single panel of the trellis and encompassing the full row width, by using a 90° view cap and excluding the lowest ‘ring’ of the sensor during the data processing using the FX2200 software provided by the manufacturer. The reading was taken with the sensor approximately 80 cm below the cordon and the 90° view facing along the row.

The VitiCanopy measurements used the frontal camera of an iPad mini (Apple Inc., Cupertino, CA, USA), positioned 80 cm below the vine cordon, close to the ground. A total of 86 LAI-2000 measurements *vs.* image analysis from VitiCanopy were compared using linear regression.

#### 2.7.3. VitiCanopy *vs.* Planimetric and Gravimetric Method

Vines (*n* = 12) were randomly selected in a Shiraz vineyard located in the McLaren Vale Region of South Australia. Vines were trained on a vertical shoot positioned (VSP) trellis with a cordon height of 100 cm from the ground. Images were obtained at 90 (*n* = 2), 80 (*n* = 3), 70 (*n* = 3), 60 (*n* = 2) and 50 (*n* = 2) cm from the cordon from each plant using the front camera of an iPad 4 (Apple Inc., Cupertino, CA, USA). At the beginning, each vine’s whole canopy was imaged (100%) and then three consecutive stripping of leaves was performed to reduce LAI until only the bare cordon was left visible. About 30% of the canopy was removed with each leaf stripping. Stripping was performed by manually plucking the leaves. Only the section of canopy that was visible in the iPad screen within each image was stripped. For this purpose, the section of visible cordon was marked using flagging tape at the cordon level for reference. Given that the field of view of the iPad front camera is about 43° (Figure 1), the length of cordon included in the image at the different distance of the camera from the cordon is reported in Table 1.

The length of cordon in the image was used to calculate the real LAI (LAI_r_) for the specific section of canopy. The latter value was then scaled up to represent leaf area (LA) in 1 m^2^ of canopy area per 1 m^2^ of soil, which is what VitiCanopy and other cover photography methods estimate and report [14,15,19]. The latter value was considered as a ground truth measurement or real LAI (LAI_r_).

To measure the leaf area removed with each stripping an adaptation of both the gravimetric and planimetric methods was used [7]. For the gravimetric method, the LA removed with each stripping was related to weight. From all the stripped material, 500 leaves were randomly selected and digital images were obtained with a reference area of 4 × 4 cm (black painted paper) included in each image. Total LA per scanned image was obtained using a customized Matlab code. The same leaves were oven dried for 48 hours at 60 °C then weighed with a precision scale to calculate the specific leaf area (SLA = LA (cm^2^)/leaf dry weight (g)) which was on average 216 cm^2^/g. The specific leaf area was then used to calculate the LA removed with each strip.

#### 2.7.4. Comparison between Real *k vs.* Fixed and Modeled *k* to Estimate LAI

Since LAI_r_ was measured directly using a combination of planimetric and gravimetric methods, a real *k* per image could be obtained by inverting Equation (4) as follows:

(7) $$k_{r}=\;-f_{c}\;\frac{\text{ln}\Phi }{{\text{LAI}}_{r}}$$

where LAI_r_ is the real LAI from the planimetric measurements and *k_r_* is the real *k* value per image obtained from the validation procedure.

The *k_r_* values were then compared to five methods to obtain a proxy and real values of *k*:

(i) Fixed value (*k* = 0.7) as reported in literature [16];
(ii) Real *k* (*k_r_*) calculated by using LAI_r_ and inverting Equation (7).
(iii) Proxy of *k* obtained by using the Lowess method to smooth the data combined with the curve-fitting tool from Matlab. The model was constructed using *f_c_* (Equation (2)) and Φ (Equation (3)) as follows: k_Smooth_ = malowess (*f_c_*, Φ), which smooths scattered data in *f_c_* and Φ. The default window size is 5% of the length of *f_f_*.
(iv) Modeled *k* obtained comparing *k_r_* with large gaps (lg), based on the fact that larger gaps will result in higher light transmission to the image acquisition point.
(v) The ratio between luminance, obtained automatically from the metadata of each image (I), using a customized code written in Matlab and the maximum luminance obtained from sky images (I_o_ = 12).

### 2.8. Statistical Analysis

Statistical analysis to compare the performance of estimated against real values of LAI and *k* included: linear regressions (y = a + bx), coefficient of determination (*R*^2^), standard error of estimates (SEE), root mean squared error (RMSE), and statistical significance of regressions with minimum *p* value ≤ 0.05 as significance criteria. All the analyses were performed using Matlab ver. 2016a (Mathworks Inc, Matick, MA. USA).

## 3. Results

### 3.1. VitiCanopy vs. MatLab

**Figure 3:**
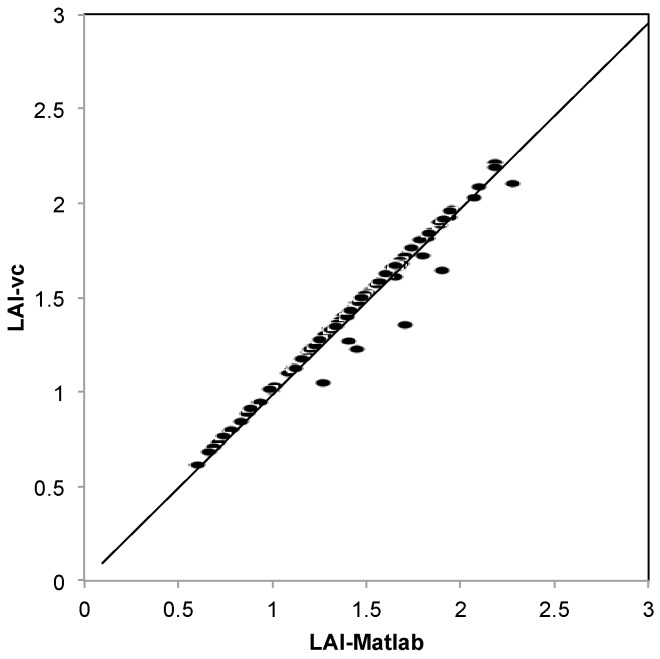
Relationship between the canopy size measured by analyzing the images using the Matlab method (LAI-Matlab) and VitiCanopy (LAI-vc). The continuous line represents the linear fitting; the dashed line represents the 1:1 relationship.

VitiCanopy, for the majority of the images analyzed, produced near identical results to those obtained with the previously developed Matlab code with the correlation producing a *R*^2^ = 0.97 and a slope b = 0.98 with minimal SEE (0.3529) and RMSE (0.056) and very high statistical significance of the regression (*p* < 0.00001) (Figure 3). The clumping index (Ω(0)) was on average 0.78 with a range from 0.61 to 1 for both methods.

### 3.2. VitiCanopy vs. Licor-2000

**Figure 4:**
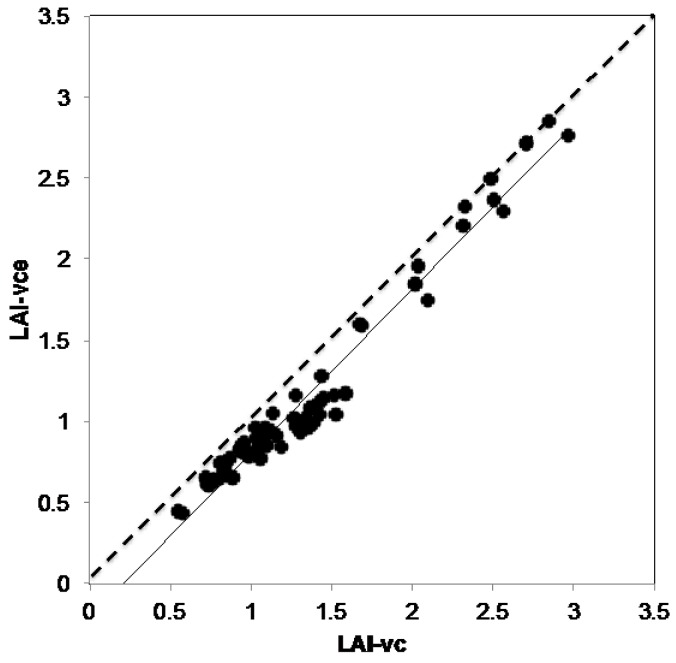
Relationship between plant area index (LAI-vc) and effective plant are index (LAIvc_e_ = LAI × Ω(0)) measured using the VitiCanopy App for three vineyards located in Langhorne Creek (SA), Hilltops (NSW) and Sunraysia (Vic) during the season 2013–14. The continuous line represents the calculated regression; the dashed line represents the 1:1 relationship.

**Figure 5:**
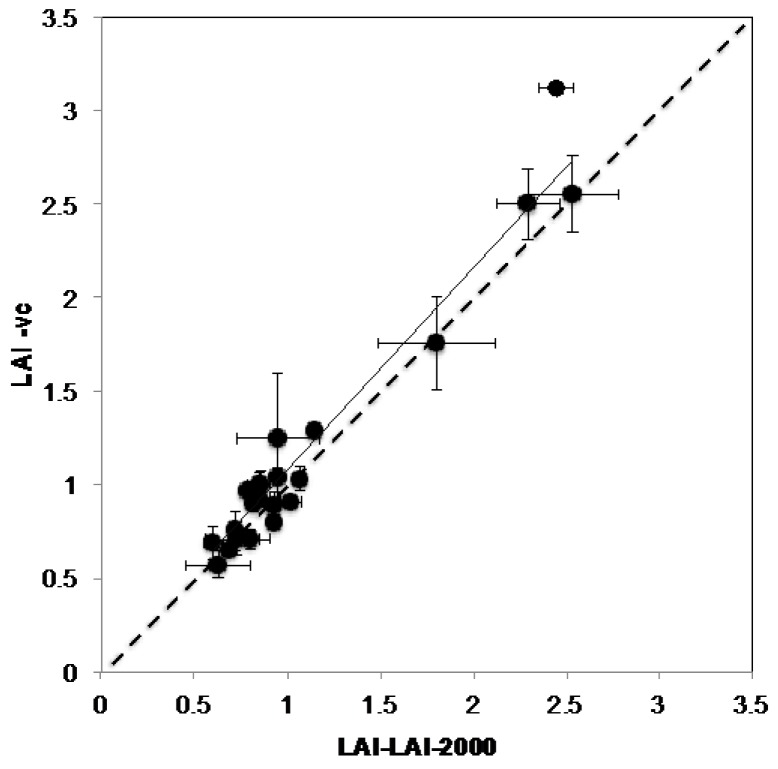
Relationship between the canopy size measured using the Licor-2000 (LAI-LAI-2000) and VitiCanopy App (LAI-vc_e_) for three vineyards located in Langhorne Creek (SA), Hilltops (NSW) and Sunraysia (Vic) during the season 2013–14. Error bars correspond to the standard error of the means. The continuous line represents the calculated regression passing through the origin (0,0), the dashed line represents the 1:1 relationship.

On average, LAI-vc ranged from 0.99 to 2.37 and Ω(0) from 0.76 to 0.98 in the three vineyards: Langhorne Creek, Hilltops and Sunraysia with a range of canopy management treatments applied at each vineyard (Table 2). The relationship between LAI-vc and LAI-vc_e_ calculated using VitiCanopy (*R*^2^ = 0.96, b = 0.25) showed that, in these vineyards, LAI-vc was an underestimation due to Ω(0) being lower than 1 and indicative of slightly clumped canopies (Table 2; Figure 4). For this reason, LAI-vc_e_ was considered a more accurate estimate of canopy size and was used for the comparisons with the Licor-2000 (Figure 5).

When the canopy size estimated with VitiCanopy (LAI-vc_e_) and using the Licor-2000 (LAI-LAI-2000) were compared, a strong and significant linear correlation between averaged values from the two methods was found (*R*^2^ = 0.95; b = 1.08; SEE = 0.51; RMSE = 0.16; *p* < 0.00001) (Figure 5).

### 3.3. VitiCanopy vs. Planimetric and Gravimetric Method

The goodness-of-fit for the correlation between the real LAI (LAI_r_) measured with the combination of planimetric and gravimetric methods and VitiCanopy, was tested by grouping the results based on the distance between the camera and the cordon from where the images were obtained (Table 3). The best results were achieved when the camera was placed at 80 cm from the cordon. However the results are accurate (*R*^2^ from 0.70 to 0.89 and slope b from 0.86 to 1.15; *p* < 0.00001 for all) with distances from 50 to 90 cm from the cordon (Table 3).

**Figure 6:**
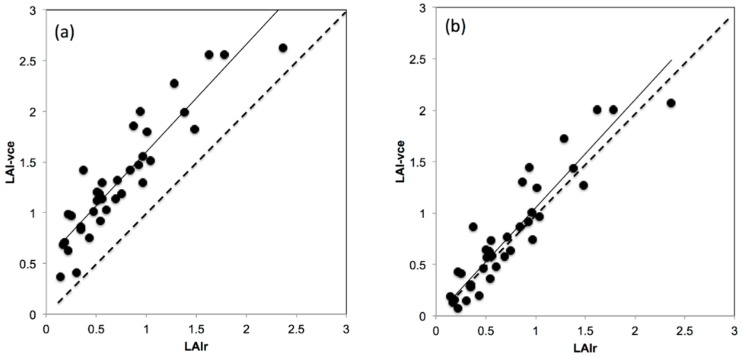
(**a**) Relationship between the leaf area index (LAI) measured by destructively removing all the leaves (LAI_r_) and using the cover photography App VitiCanopy (LAI-vc_e_) using a common light extinction coefficient (*k* = 0.7) (method i) for the cv. Shiraz on a VSP trellis system in the McLaren Vale region; (**b**) Relationship between Real LAI (LAI_r_) and VitiCanopy LAI (LAI-vc_e_) extracting the Y—intercept related to cordon and non-leaf material inclusion. For 6a and 6b, the continuous line represents the calculated regression; the dashed line represents the 1:1 relationship.

Figure 6 shows the relationships between results obtained with VitiCanopy (LAI-vc_e_) and the planimetric/gravimetric method (LAI_r_) for the whole data set. LAI-vc_e_ was used in all comparisons since the manual stripping used to reduce canopy size does create clumping; as a matter of fact, Ω(0) ranged from 0.65 to 1. The *R*^2^ obtained was 0.84 with a slope b = 1.06, SEE = 1.89 and RMSE = 0.24 at *p* < 0.00001. For the 12 vines considered, VitiCanopy overestimated the real LAI by 0.56 on average, which corresponds to the contribution of the cordon and other non-leaf material within each image. When the average LAI obtained from images of bare cordons (LAI-vc_e_ = 0.56) was subtracted to all data points, a significant improvement of the relationship was achieved in relation to the 1:1 line (*R*^2^ = 0.86; b = 1.05; SEE = 1.89; RMSE = 0.24; *p* < 0.00001).

### 3.4. Comparison between Real k vs. Fixed and Modelled k to Estimate LAI

**Figure 7:**
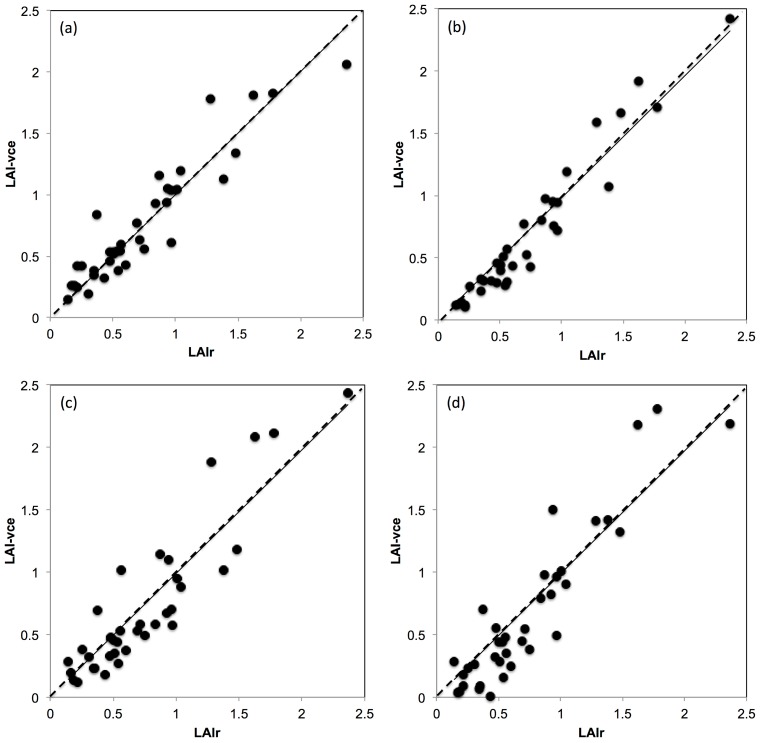
Linear regressions obtained from the comparison between real LAI (LAI_r_) and effective LAI using VitiCanopy (LAI-vc_e_) with different methods to obtain a proxy of light extinction coefficient (*k*): (**a**) obtaining a real *k* (*k_r_*) by inverting Equation (7) (method ii); (**b**) Lowess linear interpolation model based on canopy cover (*f_c_*) and porosity (Φ) (method iii); (**c**) *k* obtained from a linear regression between *k_r_* and large gaps (lg) (method iv) and (**d**) *k* obtained from the ratio between image luminance (I) and maximum luminance (Io = 12) (method v).

As seen before, Figure 6 shows the relationship between LAI_r_ and LAI-vc_e_ using a common *k* = 0.7, which corresponded to the application of method (i) to obtain a proxy of *k*. Figure 7a shows the relationship between LAI_r_ and LAI-vc_e_ by applying method (ii) with *k_r_* obtained by inverting Equation (7), which rendered highly significant statistical analysis (Table 4) as expected (*R*^2^ = 0.94; b = 0.98; SEE = 0.71; RMSE = 0.15; *p* < 0.00001). Figure 7b shows the LAI_r_ and LAI-vc_e_ relationship by using method (iii) with comparable statistical results to method (ii) (*R*^2^ = 0.87; b = 1.0; SEE = 1.11; RMSE = 0.18; *p* < 0.00001). Figure 7c shows the LAI_r_ and LAI-vc_e_ relationship by applying method (iv) using big gaps as a proxy of *k* with moderate statistical results compared to the rest (*R*^2^ = 0.83; b = 0.99; SEE = 2.13; RMSE = 0.25; *p* < 0.00001). Finally, Figure 7d shows the LAI_r_ and LAI-vc_e_ relationship by applying method (v) based on image luminosity values to obtain a proxy of *k* with also moderate statistical results (Table 4) compared to the rest (*R*^2^ = 0.86; b = 0.99; SEE = 2.04; RMSE = 0.24; *p* < 0.00001). Table 4 shows the performance of methods to obtain a proxy of *k*. The best performing methods were (i), (iii) and (iv) due to higher *R*^2^, b, SEE, RMSE and *p* values.

## 4. Discussion

### 4.1. VitiCanopy vs. MatLab

As expected, given that both VitiCanopy and Matlab use the same algorithms to calculate canopy size, the relationship between the two methods showed a good and statistically significant agreement (*R*^2^ = 0.97; b = 0.98; SEE = 0.35; RMSE = 0.056; *p* = 0.00001) (Figure 3). The discrepancy, which is a slight underestimation (3%) for the Matlab code compared to VitiCanopy for six out of 113 images, could be explained by differences in precision in the automatic luminosity thresholding, based on the blue channel, used by the two systems (computer *vs.* smartphone capabilities) [1]. When an image is taken, depending on the sky conditions, even if the sun is not directly in the image, sometimes they could present brighter sections. The algorithms used for filtering in the Matlab code compared to VitiCanopy are different and for the above-mentioned images this creates underestimations of canopy size due to loss of segmentation of images, in particular at the edges of the canopy or on over-illuminated leaves.

### 4.2. VitiCanopy vs. Licor-2000

The strong relationship found between the measurements conducted with the LAI-2000 canopy analyzer and VitiCanopy using a common *k* = 0.7 (method i) for both techniques (Figure 5) aligns with previous findings by Fuentes and collaborators [16]. These authors found a good correlation between LAI-2000 and the cover photography and Matlab methods for Chardonnay on a two-wire vertical training system in the Riverland Region of South Australia. The relationship also confirms that the use of the effective LAI output (LAI-vc_e_) rather than LAI-vc should be considered when the clumping index is lower than 1 (Figure 4). It has been previously reported [16,26] that the clumping index of grapevine is generally close to 1 since the use of training systems and the intensity of canopy management treatments applied forces the canopy into a less clumped foliar distribution growth. However, an effect of the phenological stage, vine age and training systems on the clumping index should be expected. More research is underway to understand the role of the clumping index in the estimation of LAI-vc for different training systems, varieties and microclimates.

These results demonstrate that VitiCanopy can be used within the same guidelines suggested for other instruments available in the market (e.g., the LAI-2000) with the advantages of VitiCanopy being a free tool, easy to use by trained operators and the results being obtained in real time and geo-referenced. Moreover, not only canopy size (LAI-vc) can be obtained, but also the fractions of foliage cover (*f_f_*) canopy projective cover (*f_c_*), canopy porosity (Φ) and clumping index (Ω(0)) are extracted from the canopy images. VitiCanopy also presents less restrictions for its use compared to commercial instrumentation, in particular, VitiCanopy can be used at any time of the day as long as the sun does not appear in the image. The LAI-2000 is traditionally recommended to be used either early in the morning (after predawn), late in the day (before dusk) or in overcast day conditions for greater accuracy [27]. The latter related to the scattering of blue light through the different angles of the sensor, which has been reported to induce significant underestimations [15,17]. New instrument developments however, allow the correction of the blue light scattering by using a diffuser cap allowing measurements to be taken in full sun.

### 4.3. VitiCanopy and the Effect of Distance of Images from Grapevine Cordons

Our findings suggest that the optimal distance from the cordon to take upward-looking images from grapevine canopies (Figure 1) is 70 to 80 cm (Table 2). Even though good and statistically significant relationships between LAI_r_ and LAI-vc were found for all distances from 50 to 90 cm from the cordon, users should take into account that at a distance of 50 cm between the device and the canopy, only 38 cm of cordon will be included in the image; potentially not capturing enough of the canopy variability when making the assessment. Only when conditions prevent the adoption of the 70–80 cm distance (very low training systems), a lower distance should be considered. We recommend that images not to be taken at distances less than 50 cm for reasons outlined above.

### 4.4. VitiCanopy Performance and Cordon Contribution Corrections

VitiCanopy, similar to other non-destructive methods, does not separate between leaves and other plant elements (cordon, shoots and bunches). Such output is more often reported as PAI rather than LAI [30]. However, various studies [6,15] have also found that the inclusion of non-plant material can be insignificant for the estimation of LAI in forests or, in the case of grapevines, it can overestimate LAI only by 3% in the early phenological stages and it becomes insignificant from veraison onwards [16]. It is expected that vine age (bigger cordons) and training systems (VSP *vs.* cane pruning for example) would have an effect on this. In this study, VitiCanopy overestimated LAI (Figure 6a) compared to the planimetric method applied when using a common light extinction coefficient of *k* = 0.7 (method i). When images from bare cordons, after stripping all the leaf material for the validation procedure, were analyzed, a LAI-vc_e_ on average 0.56 for cordon contribution was found (Figure 6a). When this was extracted from results as a correction factor, comparable results between LAI-vc_e_ and LAI_r_ (Figure 6b) were obtained. In practical terms, if the user requires the estimation of the cordon contribution (Figure 1), (LAI rather than PAI), this can be achieved by using VitiCanopy at dormancy, when no leaf material is present on the vines. Nevertheless, the contribution of the cordon might not be important when the aim of the assessment with VitiCanopy is to monitor the spatio-temporal growth of the vineyard’s canopy and as a comparison factor between treatments. The images used for comparison purposes between VitiCanopy and the planimetric method in this paper were obtained at flowering and it can be assumed that at this early growth stage the contribution of the cordon was still important as supported by the results obtained in Figure 6a and correction needed (Figure 6b). The latter needs to be taken into account when comparing results from VitiCanopy and other methods that exclude non-leaf material from the image analysis. Recent developments and improvements of the cover photography method applied to fruit trees base the image segmentation on the CieLab colour space rather than the Red, Blue and Green [26]. These methods have also shown that an automatic separation of leaves from bunches and other non-leaf materials can be achieved to increase accuracy to obtain absolute values of LAI. This improvement could be incorporated into future developments of VitiCanopy to avoid overestimations until veraison.

### 4.5. The Effect of Variable k on PAI Using the VitiCanopy App

From previous research conducted by applying the cover photography and Matlab analysis method on apple trees, it was concluded that PAI is very sensitive to the accurate selection of *k* [18]. The latter study demonstrated that estimating PAI using a fixed *k* produced unreliable results. Other models for *k* selection were applied and it was concluded that obtaining PAI by calculating a proxy of *k* using the fc from the same images was a better performing alternative. However, the best performing method required the calculation of a proxy of *k* (I_c_) using the ratio between the photosynthetic active radiation (PAR) measured above canopy (I_o_) and below the canopy at the location of imaging (I): I_c_ = 1 − (I/I_o_) [18].

In this paper, different methods to obtain k for grapevines trained on a VSP were tested. The results obtained comparing LAI_r_ with LAI-vc_e_ using methods i–v showed that selecting a common *k* (method i) rendered very good and statistically significant results in the calculation of LAI-vc_e_. Unlike fruit tree canopies, grapevine canopies, especially when trained on a VSP, are continuous and generally uniform. This could explain why a common *k* is so effective in the case of grapevines [16], but not for the case of apple trees [18], which are trained to develop more complex branch distribution and canopy structures to maximize light penetration to the interior of the canopy.

## 5. Conclusions

VitiCanopy offers a versatile, accurate and inexpensive tool to monitor spatial and temporal variability of canopy architecture parameters and leaf and plant area index within vineyards. VitiCanopy has the capability to stream processed information to growers in real time and the information is delivered in a simple and user-friendly way to assist management decisions. Despite the importance of canopy architecture measurements in vineyard management, due to the labor-intensive procedures required for their estimation, they are rarely measured. VitiCanopy offers the advantages of being free and easy to use; moreover, not only canopy size but also canopy porosity is measured. Viticultural practitioners and scientists can use this tool with comparable results to other technologies available in the market at a fraction of their cost and without the requirement of highly technical personnel to obtain the data, do the analyses and interpret results. Another major advantage of the cover photography method and VitiCanopy, is that images can be stored. Stored images can be revisited for future analyses if, for example, improvements of the method are made. Stored images can also help create a historical record for a specific site and hence aid in future management decisions. Furthermore, all images and results obtained using VitiCanopy are geo-referenced providing a visual guide to variation across a vineyard.

The VitiCanopy App development team from the University of Adelaide and the University of Melbourne are working on different improvements of this novel App to add more tools to assess the effects of changes in vigor, pruning weight and other canopy architecture parameters on the quality of harvested product for different crops and fruit trees. These improvements will be reflected in future version releases of the App.

## Figures and Tables

**Table 1:** Relationship between the distance of the device (iPad 4) from the vine’s cordon and the length of cordon included in the image considering the iPad4’s camera field of view of 43°.

Distance iPad-Cordon (cm)	Cordon Length in the Image (cm)
90	70
80	64
70	54
60	46
50	38

**Table 2:** Basic statistics (average and range) from results obtained using VitiCanopy to estimate canopy size for three vineyards located in Langhorne Creek (SA), Hilltops (NSW) and Sunraysia (Vic) during the season 2013–14. LAI-vc = plant area index, LAI-vc_e_ = effective plant area index (LAI-vc_e_ = LAI-vc × Ω(0)) and Ω(0) = clumping index at the 0° zenith angle.

Vineyard	LAI-vc		LAI-vc_e_		Ω(0)	
	**Average**	**Range**	**Average**	**Range**	**Average**	**Range**
Hilltops	0.99	0.72–1.44	0.85	0.57–1.28	0.85	0.76–0.90
Langhorne Creek	2.37	1.93–2.71	2.26	1.72–2.66	0.95	0.90–0.98
Sunraysia	1.30	1.16–1.39	0.97	0.90–1.04	0.75	0.70–0.78

**Table 3:** Statistics of the correlation between the results obtained with the planimetric and gravimetric method and VitiCanopy as a function of the distance between the iPad4 and the vine’s cordon. *R*^2^ = coefficient of determination, b = Slope, a = Intercept, SEE = standard error of estimates, RMSE = root mean squared error.

Distance iPad-Cordon (cm)	*R*^2^	b	a	SEE	RMSE
50	0.76	1.08	0.35	0.15	0.19
60	0.79	0.86	0.47	0.05	0.11
70	0.89	1.01	0.31	0.65	0.30
80	0.89	1.06	0.28	0.30	0.21
90	0.70	1.15	0.19	0.38	0.28

**Table 4:** Statistical results obtained for the *k*-proxy models (methods iii to v) and from LAI calculation using five different methods: using a common *k* = 0.7 (method i); obtaining a real *k* (*k_r_*) by inverting Equation (7) (method ii); Lowess linear interpolation model based on canopy cover (*f_f_*) and porosity (Φ) (method iii); *k* obtained from a linear regression between *k_r_* and large gaps (lg) (method iv) and *k* obtained from the ratio between image luminance (I) and maximum luminance (I_o_ = 12) (method v). Not applicable and not statistically significant are denoted by na and ns respectively.

Proxy of *k*	*R*^2^	b	SEE	RMSE	*p* Value
Method (i)	na; 0.86	na; 1.05	na; 1.89	na; 0.24	na; 1 × 10^−5^
Method (ii)	na; 0.94	na; 0.98	na; 0.71	na; 0.15	na; 1 × 10^−5^
Method (iii)	0.38; 0.87	1.72; 1.00	0.61; 1.11	0.13; 0.18	1 × 10^-4^; 1 × 10^−5^
Method (iv)	0.05; 0.83	1 × 10^−5^; 0.99	2.57; 2.04	0.27; 0.24	ns; 1 × 10^−5^
Method (v)	0.25; 0.86	0.09; 0.99	0.21; 2.04	0.08; 0.24	1 × 10^-3^; 1 × 10^−5^

## Supplementary Materials

VitiCanopy for iOS operating systems is available for free download at https://itunes.apple.com/au/app/viticanopy/id1042914901?mt=8. An instructional YouTube video on how to use VitiCanopy is available at https://www.youtube.com/watch?v=Eqzmuruubm8.
